# A Longitudinal Examination of the Relationship between Trauma-Related Cognitive Factors and Internalising and Externalising Psychopathology in Physically Injured Children

**DOI:** 10.1007/s10802-018-0477-8

**Published:** 2018-09-28

**Authors:** Rachel M. Hiller, Cathy Creswell, Richard Meiser-Stedman, Sarah Lobo, Felicity Cowdrey, Mark D. Lyttle, Anke Ehlers, Sarah L. Halligan

**Affiliations:** 1 Department of Psychology, University of Bath, Bath, UK; 2 School of Psychology and Clinical Language Sciences, University of Reading, Reading, UK; 3 Norwich Medical School, University of East Anglia, Norwich, UK; 4 Oxford Health NHS Foundation Trust, Cotswold House, Specialist Eating Disorder Service, Savernake Hospital, Marlborough, UK; 5 Emergency Department, Bristol Royal Hospital for Children, Bristol, UK; 6 Faculty of Health and Applied Sciences, University of West England, Bristol, UK; 7 Department of Experimental Psychology, University of Oxford, Oxford, UK

**Keywords:** Posttraumatic stress disorder, Internalising and externalising, Appraisals, Memory, Longitudinal

## Abstract

Cognitive models of posttraumatic stress disorder (PTSD) highlight maladaptive posttrauma appraisals, trauma memory qualities, and coping strategies, such as rumination or thought suppression, as key processes that maintain PTSD symptoms. Anxiety, depression and externalising symptoms can also present in children in the aftermath of trauma, yet there has been little empirical investigation of the potential relevance of posttrauma cognitive processes for such difficulties. Here, we examined whether: a) acute maladaptive cognitive processes (specifically, maladaptive appraisals, memory qualities, and cognitive coping) were associated with symptoms of PTSD, internalising, and externalising at 1-month posttrauma (T1); and b) changes in these cognitive processes predicted symptom change at a follow-up assessment 6 months later (T2). We recruited 132 6–13 year old children and their parents from emergency departments following the child’s experience of an acute trauma. Children self-reported on their maladaptive appraisals, trauma-memory and cognitive coping strategies, along with symptoms of PTSD, anxiety and depression. Parents also rated children’s internalising and externalising symptoms. We found each cognitive process to be robustly associated with PTSD and non-PTSD internalising symptoms at T1, and change in each predicted change in symptoms to T2. Maladaptive appraisals and cognitive coping were unique predictors of children’s posttrauma internalising. Effects were partially retained even controlling for co-occurring PTSD symptoms. There was less evidence that trauma-specific cognitive processes were associated with externalising symptoms. Findings suggest aspects of cognitive models of PTSD are applicable to broader posttrauma psychopathology, and have implications for how we understand and target children’s posttrauma psychological adjustment.

**Electronic supplementary material:**

The online version of this article (10.1007/s10802-018-0477-8) contains supplementary material, which is available to authorized users.

Following a young person’s experience of trauma, they are at risk of developing a range of poor mental health outcomes, of which the most widely studied is posttraumatic stress disorder (PTSD; Alisic et al. 2014; Hiller et al. 2017). While elevated PTSD symptoms (PTSS) can be common in the posttrauma period, for many young people initial symptoms will naturally recede without the need for formal support (Hiller et al. 2017; Le Brocque et al. 2009), with 10–20% experiencing more chronic distress (Alisic et al. 2014; Hiller et al. 2017). However, PTSD is just one potential adverse psychological outcome following trauma – other internalising difficulties (e.g., anxiety, depression) and externalising difficulties (e.g., attention and behaviour problems) can also be elevated in trauma-exposed young people (e.g., Pine and Cohen 2002; Scheeringa and Zeanah 2008). While relatively robust empirical evidence has identified key psychological mechanisms that contribute to the maintenance of chronic PTSS (e.g., Ehlers and Clark 2000; Trickey et al. 2012), the role of these processes in relation to other posttrauma psychological outcomes has been little studied. Such investigation is important for understanding the extent to which targeting trauma-related psychological processes in intervention is likely to address other adverse mental health outcomes, beyond PTSD. It also has theoretical implications, especially for understanding whether current models specific to the maintenance of PTSD may more accurately represent models of children’s broader posttrauma mental health.

Theories of the development and maintenance of PTSD highlight several key psychological processes that may lead to, or maintain, PTSS (Brewin et al. 1996; Ehlers and Clark 2000; Foa et al. 1989; Meiser-Stedman 2002). For example, Ehlers and Clark’s cognitive model of PTSD (Ehlers and Clark 2000) identifies three core posttrauma processes that maintain PTSD, particularly by contributing to a sense of current threat. First, the individual makes strong negative appraisals of the event or their own responses (e.g., “I’ll never get over what happened”). Second, the trauma memory is proposed to be encoded in such a way that it is particularly prone to retrieval via direct triggering through matching cues and has ‘flashback’ like properties (e.g., due to a high level of sensory content, and limited contextual or semantic encoding). Third, engagement in maladaptive coping strategies is hypothesised to maintain maladaptive appraisal and trauma memory properties (e.g., due to cognitive avoidance), and directly exacerbate symptoms (e.g., due to counterproductive attempts to suppress trauma memories).

The empirical literature, including a number of meta-analytic reviews, provides strong support for the central role of posttrauma cognitive processes in the maintenance of traumatic stress. For example, meta-analytic reviews of the child and adolescent trauma literature have concluded that the use of thought suppression (a maladaptive cognitive coping strategy) is moderately to strongly positively associated with PTSS severity (Trickey et al. 2012), and that there is a strong association between maladaptive appraisals and PTSS (Mitchell et al. 2017; Trickey et al. 2012). The role of trauma-related memory quality is more well-established in the adult PTSD field (Brewin 2014), although there is some evidence for a significant association between sensory-laden trauma memories and higher PTSS among children following trauma (Salmond et al. 2011; Meiser-Stedman et al. 2007a; McKinnon et al. 2017). Moreover, trauma-focused cognitive behavioural therapy (tf-CBT), the recommended first-line treatment for PTSD in children (Cohen et al. 2000; Perrin et al. 2000), specifically targets the reduction of maladaptive appraisals, unhelpful coping strategies, and problematic memory qualities.

While trauma-related cognitive processes are relatively well-established in the development and maintenance of PTSS, there has been less extensive examination of their potential role in relation to broader posttrauma psychopathology. This omission is important, as other internalising difficulties such as anxiety and depression, as well as externalising difficulties such as behaviour problems, are also all relatively common sequelae of trauma exposure in young people, and are commonly comorbid with PTSD (e.g., Cénat and Derivois 2015; Scheeringa and Zeanah 2008; Scheeringa 2015). Notably, trauma-related maladaptive appraisals have been found to be significantly cross-sectionally associated with posttrauma anxiety and depression following sexual assault (e.g., Mannarino and Cohen 1996); with internalising and externalising symptoms in a community sample of children and teens (Liu and Chen 2015); and with depression, but not carer-reported externalising, following maltreatment (Leeson and Nixon 2011). This is consistent with the centrality of maladaptive, dysfunctional or distorted cognitions about the self, others, and/or world, to models of child depression (e.g., Birmaher et al. 1996), anxiety (e.g., Ellis and Hudson 2010; Rapee and Heimberg 1997), and conduct problems (e.g., Dodge and Pettit 2003). The role of posttrauma coping and memory quality in relation to broader psychopathology has been less well established. However, rumination has been associated with non-trauma specific depression in children (e.g., see Hitchcock et al. 2014), while avoidant coping is central to the maintenance of a broad range of child internalising difficulties (e.g., Murray et al. 2009). Equally, researchers have highlighted the presence of negative intrusive memories in depression and in anxiety disorders (e.g., Hackmann and Holmes 2004; Patel et al. 2007)

The primary aim of the current study was to investigate the potential role of key trauma-related cognitive processes in relation to a range of psychological outcomes in children, in a longitudinal study of an acute-trauma exposed sample (Hiller et al. 2017). We examined whether: a) trauma related appraisals, memory qualities, and maladaptive coping strategies showed cross-sectional associations with anxiety, depression and externalising symptoms, measured 1-month posttrauma, as well as with symptoms of PTSD; and b) whether changes in cognitive processes from 1 to 7 months were associated with internalising and externalising severity across this period. Given that there is overlap between symptoms of PTSD and those of depression and anxiety problems, we also examined whether posttrauma cognitive mechanisms made a unique contribution to the prediction of anxiety, depression, and/or externalising symptoms once levels of PTSS were controlled for. Although a particularly stringent analysis, this allowed for stronger inferences in relation to whether posttrauma psychological processes are relevant intervention targets even in the absence of significant PTSS.

## Method

### Participants

Participants were 132 children aged 6–13 years old, and their caregivers, recruited following the child’s involvement in a trauma and subsequent attendance at the emergency department (ED). Participants were recruited between April 2014 and January 2016, from four EDs in the UK, as previously reported (Hiller et al. 2017). Exclusion criteria were: significant learning difficulty or neurodevelopmental disorder; organic traumatic brain injury; suspicion that the injury was caused by the young person themselves or their carer. Thirty-nine percent of all potentially eligible participants were recruited to the final study (main reason for non-participation was that families could not be contacted). We found no evidence that study participants differed from the eligible population. Complete recruitment details are presented in Hiller et al. 2017, while the recruitment flow-chart is available in supplementary materials (S1). Index traumas were: motor vehicle accident (*n* = 68, 52%), fall from a height (*n* = 25, 19%), significant bicycle accident (*n* = 9, 7%), acute medical episode (*n* = 10, 8%; e.g., acute anaphylaxis), sport injury (*n* = 6, 5%), and assault (*n* = 3, 2%) or other event (*n* = 17, 13%, e.g., house fire, dog attack, near drowning, sports injury).

### Procedure

ED staff initially approached families and obtained permission for the research team to make contact in order to confirm eligibility and recruit them to the study. Informed consent was provided by the caregiver, while informed assent was provided by the young person. Data presented in the current paper are from home assessments completed independently by parents and children at 2–6 weeks post-ED attendance (T1) and 6-months later (T2; i.e., 7-months posttrauma). Dyads received GBP20 at each time point, as a thank you for their time. An additional 3-month postal questionnaire assessment also took place, as reported in Hiller et al. 2017. Ninety-six percent of the sample were retained at the 6-month assessment.

### Measures

Demographic and trauma-related information were obtained from ED notes and parent interview. Objective trauma severity was assigned using the Manchester Triage System (nurse rating of urgency of care), ranging from 1 = *immediate care required* to 4 = *standard* (i.e., less urgent). Key study indices were then obtained through child and parental questionnaires, completed at both T1 and T2. Specifically, we utilised child self-report measures of cognitive processes, PTSS, anxiety and depressive symptoms. By contrast, we used parental report as our main measure of child externalising, as is recommended in the literature (Stanger and Lewis 1993).

#### Maladaptive Trauma Appraisals

Children completed the Children’s Posttraumatic Cognitions Inventory (CPTCI), a 25-item self-report measure suitable for children as young as 6 years old (Meiser-Stedman et al. 2009). The CPTCI measures appraisals relating to: (i) permanent and disturbing change (e.g., “My reactions since the event mean I have changed for the worse”) and (ii) being a fragile person in a scary world (e.g., “Anyone could hurt me”). Young people rate their agreement with each statement from 1 = *don’t agree at all* to 4 = *agree a lot*, with the total score providing an overall index of maladaptive appraisals (ranging from 25 to 100; α = 0.93).

#### Posttrauma Coping

Children completed the Child Posttrauma Coping Questionnaire (CPCQ), an 11-item self-report scale created for this study based on cognitive coping items used in previous research (Ehlers and Clark 2000; Ehlers et al. 2003; Stallard 2003). The measure includes 5 items on rumination (e.g., “I can’t stop thinking if only the event hadn’t happened to me”) and 6 items on thought suppression (e.g., “I’ve tried to keep any memories of what happened out of my head”), each rated on a four-point scale from 0 = *not at all or only one time* to 3 = *a lot of the time*, and summed to yield a total score (between 0 and 30). The measure shows good internal consistency (α = 0.89) and validity against a measure of child PTSS symptoms (Hiller et al. 2017).

#### Trauma Memory Quality

Children completed an adapted version of the Trauma Memory Quality Questionnaire (TMQQ; Meiser-Stedman et al. 2007a). This 18-item measure covers the original scale’s 11-items on the sensory quality of the trauma-memory and sense of “nowness” (e.g., “My memories of the event are mostly pictures of images”; “When I think about the frightening event I can sometimes smell things that I smelt when the frightening event happened”), and an additional 7-items on disorganised memories (e.g., “I get mixed up about what order things happened in during the frightening event”), adapted from an equivalent adult scale (e.g., Halligan et al. 2003). Items are rated on a scale from 1 = *disagree a lot* to 4 = *agree a lot* and summed to yield a total score (range, 18–72). Internal consistency of the 18-item scale was α = 0.86, compared to α = 0.80 for the original 11-item scale.

#### PTSS

Children completed the child self-report version of the PTSD Reaction Index (PTSD-RI), which has established reliability and validity for children as young as 6 years old (Steinberg et al. 2004). The PTSD-RI assesses 17 DSM-IV-TR PTSD symptoms. Responses to each item are rated on a 5-point Likert scale, ranging from 0 = *none of the time* to 4 = *most of the time,* and yielding a total symptom score as the main outcome (ranging from 0 to 68; α = 0.89).

#### Anxiety and Depression

Children completed the 25-item short version of the Revised Child Anxiety and Depression Scale (RCADS; Chorpita et al. 2000; Ebesutani et al. 2012), a widely used, self-report measure, validated with children aged 7 years and over (Ebesutani et al. 2012). Items are rated on a 4-point scale ranging from 0 = *never* to 3 = *always*, measuring 15 symptoms of anxiety (e.g., “I worry about being away from my parents”; total score range 0–45; α = 0.89) and 10 depressive symptoms (e.g., “I feel worthless”; total score range 0–30; α = 0.86).

#### Parent-Report on Child Internalising and Externalising

Parents completed the Strengths and Difficulties Questionnaire (SDQ) parent-report version, a widely used measure of children’s emotional and behavioural difficulties, suitable for reporting on children as young as 4 years of age (Goodman 1997). Parents rate items from 0 = *not true* to 2 = *certainly true*. We utilised the 10-item externalising subscale which comprises items covering hyperactivity and conduct problems (e.g., “Often has temper tantrums or hot tempers”, “Easily distracted”; total score range 0–20; α = 0.76); and the 10-item internalising scale, which assesses emotional and peer problems (e.g., “Many worries, often seems worried”; “Picked on by other children”; total score range 0–20; α = 0.80). The SDQ was added to the T1 assessment during the course of the study; consequently, T1 data is only available for 106 participants.

### Data Analytic Plan

Data were analysed using IBM SPSS Statistics for Windows v22 (2013, IBM Corp., Armonk, NY). All mental-health outcome measure scores were positively skewed, so a square-root transformation was applied. As most 6-month data remained significantly skewed, associations were checked against non-parametric tests (Spearman’s rho), with any discrepancies noted in Table 2. Our primary aims were to explore (1) whether the three cognitive processes (appraisals, memory, coping) would be associated with acute (1-month, T1) symptom severity, and (2) whether change in these processes from 1 to 7 months posttrauma (T1 – T2) would be associated with change in symptoms over this period. In all cases, we also examined whether cognitive processes would continue to be significantly associated with symptom outcomes, even after controlling for PTSS. We used preliminary correlational analyses to establish which cognitive variables showed significant univariate associations with mental health outcomes (*p* < .05), for inclusion in subsequent regression analyses. To index T1-T2 change in cognitions we generated residual change scores via linear regressions. Next, we ran separate linear regressions to explore which of the three cognitive processes (measured at T1) most strongly predicted 1-month symptom severity (T1 PTSS, anxiety, depression, parent-reported internalising and externalising), when they were examined simultaneously (i.e., entered into a single step in the regression). For non-PTSS outcomes, additional models tested for predictive effects after controlling co-occurring PTSS. Next, to explore the longitudinal association between cognitions and symptoms, we looked at whether change in cognitive processes was associated with change in symptoms, by running linear regression models with T2 symptoms as the dependent variable, whilst controlling for T1 symptoms in the model. In particularly conservative analyses, these regressions for non-PTSS outcomes were re-run also controlling for change in PTSS (residual change scores), to explore whether change in the cognitive processes would continue to predict depression/anxiety/internalising/externalising symptoms change, even above what is explained by PTSS. Age, sex and triage (a marker of objective trauma severity) were explored as potential covariates.

Overall there was little missing data (maximum missing data was less than 10%). The only exception to this was the T1 SDQ, which was added after the study begun and was missing for 20% of the sample. In all cases data were missing completely at random. As a sensitivity analysis, to account for missing data, key analyses were run using multiple imputation with 50 iterations and predictive mean matching. The pattern of results was the same and completer-only data are presented here.

## Results

### Descriptive Information

Sample characteristics are presented in Table 1. The sample comprised 132 children (62% boys), aged 6–13 years old, and their participating parent (90% mothers). Child age, sex, and triage category were assessed as potential covariates. The child’s age was negatively associated with PTSS at both time points (*r* > −0.18, *p* > 0.04). Sex was significantly associated with initial anxiety (*r* = 0.21, *p* = 0.04) and depression (*r* = 0.20, *p* = 0.049) symptom severity, with higher symptom scores associated with being female. Triage ratings were not significantly associated with any measures of mental health (all *ps* > 0.13). Age and sex were controlled in all subsequent analyses.

**Table 1:** Descriptive information

Demographic characteristics	Statistic (*N* = 132)
Parent characteristics	
Age in years (*M [SD]*)	39.7 (7.0)
Proportion mothers	119 (90%)
Proportion married or cohabiting	97 (74%)
Education status: School until 16 years or younger	36 (27%)
Further education	50 (38%)
Higher education	46 (35%)
Child characteristics	
Age in years, *M (SD*)	9.87 (1.8)
Male	82 (62.1%)
Ethnicity – Caucasian	121 (91.7%)
Triage category	
1 (immediate attention required)	61 (46%)
2 (very urgent)	29 (22%)
3 (urgent)	26 (20%)
4 (less urgent)	16 (12%)
Days in hospital (Min – Max, *M* [*SD*])	0–28, 2.64 (4.83)
Days of school missed^a^ (Min – Max, *M* [*SD*])	0–28, 5.52 (5.98)
Proportion requiring ambulance/helicopter	90 (70%)
Proportion with head injury	33 (25%)

^a^Days of school missed represents days missed prior to their first assessment

Paired samples *t*-tests indicated that children’s self-reported symptoms significantly reduced from 1-month to 6-months across all measures (*M* and *SDs* presented in Table 2; PTSS, *p* < 0.001; Anxiety, *p <* 0*.*001; Depression, *p* < 0.001). By contrast, there was no significant change in parent-report of child internalising (*p* = 0.75) or externalising symptoms (*p* = 0.47).

**Table 2:** Bivariate correlation matrix for associations between study variables

	1.	2.	3.	4.	5.	6.	7.	8.	9.	10.	11.	12.	13.
1-mo Processes													
1. Appraisals													
2. Coping	0.61**												
3. Memory	0.59**	0.63**											
1-mo Outcomes													
4. PTSS	0.68**	0.79**	0.75**										
5. Anxiety	0.74**	0.68**	0.61**	0.74**									
6. Depression	0.70**	0.69**	0.58**	0.79**	0.84**								
7. Internalising	0.37**	0.23*^a^	0.22*	0.25**	0.18^+^	0.24*							
8. Externalising	0.29**	0.09	0.03	0.21^+^	0.24*	0.29**	0.51**						
6-mo Outcomes													
9. PTSS	0.59**	0.54**	0.39**	0.62**	0.68**	0.68**	0.21*	0.30**					
10. Anxiety	0.62**	0.43**	0.35**	0.54**	0.67**	0.64**	0.12	0.29**	0.75**				
11. Depression	0.61**	0.45**	0.30**	0.54**	0.67**	0.71**	0.25*	0.34**	0.79**	0.84**			
12. Internalising	0.28**	0.13^a^	0.17^+^	0.29**	0.36**	0.41**	0.53**	0.55**	0.49**	0.39**	0.55**		
13. Externalising	0.23*	−0.003	0.06	0.13	0.26*	0.21^+^	0.33**	0.61**	0.29**	0.27*	0.39**	0.67**	
*M* (*SD*)	40.20 (13.47)	14.52 (8.86)	37.51 (10.31)	18.84 (13.54)	9.65 (8.40)	6.30 (6.67)	3.84 (3.71)	6.01 (3.89)	12.86 (11.88)	6.67 (7.18)	4.08 (4.82)	3.72 (4.05)	6.27 (4.79)

^a^Using Spearman’s rho, 1-mo parent-report internalising was no longer significantly correlated with coping (*r*_*s*_ = 0.20, *p* = 0.06) but 6-mo parent-reported internalising was significantly correlated with coping (*r*_*s*_ = 0.26, *p* = 0.02). Coping was retained in the regression models. There were no other discrepancies between parametric (Bivariate) and non-parametric (Spearman’s Rho) tests

^+^*p* < 0.10, *p < 0*.*05, **p < 0.001. PTSS = child-reported posttraumatic stress symptom severity. ‘Internalising’ and ‘Externalising’ based on parent-report on the SDQ, all other questionnaires were child self-report. Possible ranges of scores for each measure are presented in the Method

### Posttraumatic Stress Symptoms

Significant bivariate correlations were present between all cognitive processes (i.e., appraisals, trauma memory, cognitive coping) and PTSS at T1, and change in each process was significantly associated with change in PTSS scores from T1 to T2 (see correlation matrices in Tables 2 and 3). In a regression model controlling for age and sex, the combined T1 cognitive processes explained 72% of variance in T1 PTSS, *F*(3,114) = 115.98, *p* < 0.001, with appraisals (*β* = 0.30, *p* < 0.001), memory (*β* = 0.33, *p* < 0.001) and coping (*β* = 0.41, *p* < 0.001) each being unique predictors in the model. We next tested whether change in cognitive processes predicted change in PTSS, by examining prediction of T2 PTSS by cognitive change scores, in a regression model controlling for T1 PTSS (as well as age, sex). Combined cognitive processes explained 41% of variance, *F*(3,102) = 43.11, *p* < 0.001, and each remained a unique predictor in this longitudinal model (appraisals *β* = 0.43, *p* < 0.001; memory *β* = 0.15, *p* = 0.034; coping *β* = 0.17, *p* = 0.018).

**Table 3:** Bivariate correlations between residual change scores

	1.	2.	3.	4.	5.	6.	7.
1. Appraisals							
2. Coping	0.59**						
3. Memory	0.55**	0.50**					
4. PTSS	0.77**	0.56**	0.56**				
5. Anxiety	0.63**	0.51**	0.41**	0.67**			
6. Depression	0.61**	0.47**	0.36**	0.67**	0.72**		
7. Internalising	0.25*	0.33**	0.16	0.28**	0.31**	0.40**	
8. Externalising	0.22*^a^	0.19	0.08	0.12	0.13	0.27**	0.52**

^a^Using Spearman’s Rho, change in appraisals was not significantly associated with change in externalising (*r =* 0.12, *p* = 0.29). There were no other discrepancies between parametric (Bivariate) and non-parametric (Spearman’s Rho) tests

*p < 0.05, ** *p* < 0.01. All scores are 1–6 month residual change scores. Internalising and Externalising based on parent report, all other scores based on child self-report

### Anxiety Symptoms

Each cognitive process was significantly positively correlated with child reported anxiety at T1, and change in each process was associated with equivalent change in anxiety scores from T1 to T2 (see Tables 2 and 3). In a regression model controlling for age and sex, combined T1 cognitive processes explained 63% of variance in T1 anxiety scores, with each being a unique predictor (see Table 4). When additionally controlling for T1 PTSS, the combined cognitive processes still explained 12% of variance in T1 anxiety, and maladaptive appraisals and coping remained unique predictors (see Table 4).

**Table 4:** Results of linear regressions for T1 cognitive predictors of T1 symptoms, after controlling for age and sex (Model 1) and T1 PTSS (Model 2)

	Model 1	Model 2
Child Report		
Anxiety	*R*^*2*^ = 0.63*, F*(3,114) = 77.6**	*R*^*2*^ = 0.12, *F*(3,113) = 14.7**
Appraisals	0.51**	0.45**
Memory	0.16*	0.10
Coping	0.28**	0.21**
Depression	*R*^*2*^ = 0.59*, F*(3,114) = 62.6***	*R*^*2*^ = 0.05, *F*(3,113) = 6.04***
Appraisals	0.44**	0.29**
Memory	0.19*	0.06
Coping	0.30**	0.15^+^
Parent Report		
Internalising	*R*^*2*^ = 0.15*, F*(3,92) = 5.4**	*R*^*2*^ = 0.06, *F*(3,91) = 2.2^+^
Appraisals	0.36**	0.34*
Memory	0.06	0.03
Coping	−.01	−0.05
Externalising	*R*^*2*^ = 0.07*, F*(1,94) = 7.5**	*R*^*2*^ = 0.07*, F*(1,93) = 4.2
Appraisals	0.28**	0.28*

*R*^2^ and *F* statistics relate to the change in model fit following the inclusion of cognitive variables, controlling for age and sex of the child (Model 1) and additionally for PTSS (Model 2)

** *p ≤* 0.01, *p < 0.05, ^+^*p* < 0.10

We next tested whether change in cognitive processes significantly predicted change in anxiety from T1 to T2, by controlling for T1 anxiety scores (and age and sex) in a regression model with T2 anxiety as the dependent variable. In combination, cognitive change scores explained 28% of variance in this model, with change in maladaptive appraisals and cognitive coping, but not trauma-memory, uniquely predicting change in anxiety symptoms across the 6-months (see Table 5). When additionally controlling for change in PTSS, combined cognitive processes remained a significant predictor of anxiety, explaining 3% of variance, although no individual cognitive processes uniquely predicted anxiety change (Table 5).

**Table 5:** Results of linear regressions analyses of longitudinal data examining change in cognitive processes as a predictor of symptom change (Model 1), including when controlling for change in PTSS (Model 2)

	Model 1	Model 2
Child Report		
Anxiety	*R*^*2*^ = 0.28, *F*(3,102) = 22.4**	*R*^*2*^ = 0.03, *F*(3,101) = 3.0*
Appraisals	0.38**	0.19^+^
Memory	0.02	−0.03
Coping	0.20*	0.15^+^
Depression	*R*^*2*^ = 0.28, *F*(3,102) = 23.3***	*R*^*2*^ = 0.03, *F*(3,101) = 2.4^+^
Appraisals	0.41**	0.17^+^
Memory	0.003	−0.06
Coping	0.19*	0.12
Parent Report		
Internalising	*R*^*2*^ = 0.10, *F*(2,81) = 6.6**	*R*^*2*^ = 0.03, *F*(2,80) = 2.2
Appraisals	0.03	−0.16
Coping	0.30*	0.24*
Externalising	*R*^*2*^ = 0.02*, F*(1,82) = 3.4^+^	*R*^*2*^ = 0.01*, F*(1,81) = 2.0
Appraisals	0.16^+^	0.19

*R*^2^ and *F* statistics relate to the change in model fit following the inclusion of cognitive variables. For the change models, we examined whether T1 to T2 residualized change scores for cognitive processes predicted T2 symptom severity, controlling for age, sex and T1 symptoms (Model 1), and then once also controlling for residualized change in PTSS (Model 2)

** *p ≤* 0.01, *p < 0.05, ^+^*p* < 0.10

### Depressive Symptoms

Each cognitive process showed significant positive associations with T1 depression and with change in depression from T1 to T2 (see Tables 2 and 3). In a regression analysis, combined T1 cognitive processes explained 59% of variance in T1 depression, *F*(3,114) = 62.64, *p* < 0.001, with all three processes being significant independent predictors (see Table 4). When additionally controlling for T1 PTSS, cognitive processes explained 5% of the variance in depression scores, and maladaptive appraisals remained uniquely associated with depression severity (Table 4).

Next, after controlling for initial depression severity (and age and sex), change in cognitions from T1 to T2 significantly predicted T2 depression scores, explaining 28% of variance. Here change in maladaptive appraisals and cognitive coping both uniquely predicted depression severity. However, after additionally controlling for change in PTSS scores, change in cognitive processes no longer significantly predicted changes in depression (see Table 5).

### Parent-Reported Child Internalising Symptoms

Each cognitive process was correlated with parent-reported child internalising scores at T1, but only maladaptive appraisals and coping (not memory) were significantly associated with change in symptoms from T1 to T2 (see Table 3). At T1, in combination, the acute cognitive processes explained 15% of variance in parent-reported internalising, with maladaptive appraisals being a significant unique predictor (see Table 4). After additionally controlling for T1 PTSS, cognitive processes no longer explained significant variance in T1 parent-report internalising, although maladaptive appraisals remained a unique significant predictor (Table 4).

As only change in appraisals and coping from T1 to T2 were significantly associated with change in internalising severity (Table 3), only these processes were examined further in regression models. In a model controlling for initial internalising scores, cognitive change scores in combination explained a significant 10% of variance in 6-month internalising problems. Here, change in the child’s cognitive coping was uniquely associated with change in internalising severity, but appraisal change was not (Table 5). After controlling for change in PTSS, combined cognitive processes no longer explained significant variance, although change in cognitive coping remained a unique significant predictor of change in parent-report internalising (Table 5).

### Parent-Reported Child Externalising Symptoms

Only maladaptive appraisals showed significant bivariate associations with externalising symptoms at either T1 or with change at T2, and was considered in regression models (see Tables 2 and 3). After controlling for age and sex, initial maladaptive appraisal scores were significantly associated with parent-reported externalising symptoms at T1, explaining 7% of variance. This effect remained significant after controlling for concurrent 1-month PTSS, explaining 4% of variance in parent-reported child externalising symptoms (see Table 4). However, after controlling for initial externalising symptoms (and age and sex), **c**hange in maladaptive appraisals from T1 to T2 was not significantly predictive of T2 externalising (Table 5).

## Discussion

We examined trauma-related psychological processes (appraisals, coping, memory) as potential predictors of children’s broader psychological outcomes following exposure to acute trauma. We replicated findings that all three processes explain unique variance in acute PTSS and reductions in PTSS over time. We also found robust evidence that children’s posttrauma cognitive processes were cross-sectionally and longitudinally associated with non-PTSD internalising problems, based on both self- and parent-report. These associations with wider child internalising symptoms were particularly reliable for child-reported maladaptive appraisals and maladaptive cognitive coping strategies (i.e., thought suppression and rumination); and were partially retained even after levels of PTSS were controlled for. There was less evidence for the role of trauma related cognitive processes in predicting externalising difficulties.

Our findings add to the limited literature showing trauma-related cognitive processes are important for children’s broader mental health following trauma. In particular, it builds on cross-sectional studies that have demonstrated associations between trauma-related negative appraisals and broader psychopathology (e.g., Leeson and Nixon 2011; Liu and Chen 2015). The current study found that maladaptive appraisals, coping and trauma memory quality at 1-month posttrauma were each moderately to strongly correlated with children’s self-reported depression and anxiety symptoms, and showed slightly smaller associations with parent-reported child internalising symptoms. Each cognitive process explained unique variance in child reported non-PTSD internalising symptoms at 1-month posttrauma, whereas negative appraisals were the only independent predictor of parent-reported internalising at this timepoint. Longitudinal analyses examining change in cognitive processes over time found a similar pattern of results. Together, changes in maladaptive cognitive processes explained a relatively large 28% of variance in change in anxiety and depression across a 6-month follow-up, and a smaller 10% of variance in change in parent-reported internalising problems. Changes in appraisals and coping were identified as uniquely associated with symptom change when cognitive processes were examined simultaneously as predictors.

Although not the main focus of the current study, we also found robust longitudinal evidence that appraisals, memory and maladaptive coping strategies each explain unique variance in children’s PTSS, consistent with cognitive models (e.g., Ehlers and Clark 2000). In particularly stringent analyses, we examined whether cognitive processes could predict broader internalising symptoms when controlling for co-occurring PTSS. This substantially reduced the variance explained by cognitive factors, which is unsurprising given the strong correlation and symptom overlap between PTSS, anxiety and depression. Nevertheless, even after controlling for concurrent PTSS we still found that maladaptive appraisals (e.g., “the world is unsafe”, “I’ll never get over what happened”) and cognitive coping (i.e., thought suppression, rumination), remained cross-sectionally associated with children’s non-PTSD internalising at 1-month posttrauma. Moreover, in longitudinal analyses, reductions in maladaptive cognitive coping over time continued uniquely to predict change in parent-reported child internalising problems, while the combined cognitive processes significantly predicted reductions in child reported anxiety. This indicates that associations are unlikely to be secondary to PTSD symptoms, but rather that cognitive processes potentially make a direct contribution to children’s wider internalising problems following trauma.

It is notable that whereas negative appraisals and maladaptive coping strategies were relatively consistently associated with children’s anxiety and depression, there was less robust evidence for the role of trauma-related memory qualities in relation to longer-term broader psychopathology. Thus, although trauma memory quality showed a clear pattern of bivariate associations with child internalising symptoms, it did not emerge reliably as a unique predictor of distress over and above other processes, particularly in longitudinal models; and there was no evidence that memory quality predicted wider internalising problems once PTSS was controlled for. Caution is warranted when interpreting these null findings, due to the strong overlap between all three processes and sets of symptoms. Nonetheless, in this case we cannot rule out the possibility that associations with anxiety and depression are accounted for by the co-occurrence of PTSD symptomatology. Whereas negative appraisals and maladaptive coping strategies are widely implicated in models of anxiety (e.g., thought suppression) and depression (rumination), trauma-memory quality may be a unique driver of traumatic stress. This is consistent with theoretical perspectives that highlight traumatic memories as a key defining feature of PTSD versus other emotional disorders, both phenomenologically and in terms of underlying neural processes (e.g., Elzinga and Bremner 2002).

Overall, our evidence highlights the potential utility of considering trauma-related maladaptive coping strategies and appraisals as potential treatment targets in depressed or anxious youth who present following trauma exposure. That the same processes that maintain PTSS also contribute to non-PTSD internalising may also explain why many CBT trials targeting PTSD see concurrent reductions in internalising comorbidities (e.g., Deblinger et al. 2011; Goldbeck et al. 2016; Smith et al. 2007). Our findings suggest that this change in broader psychopathology likely particularly results from the targeting of children’s maladaptive appraisals and coping strategies. While our findings suggest that a robust focus on appraisals and maladaptive coping may be particularly clinically useful when young people present with internalising problems linked to trauma, replication is required before drawing clinical conclusions.

We found substantially less evidence that posttrauma cognitive processes contribute to child externalising symptoms. Our findings build on Liu and Chen’s (2015) study with a community-sample of adolescents, replicating a cross-sectional association between appraisals and externalising, but additionally suggest that changes in trauma-related appraisals do not drive longer-term adjustment in terms of externalising behaviour. We found no evidence that trauma memory quality or maladaptive cognitive coping relate to children’s posttrauma externalising symptoms. The suggestion that these psychological processes may be less relevant to the maintenance of child externalising is consistent with some findings from treatment studies. For example, Deblinger et al. (2011) found that externalising was more effectively targeted in a treatment that had a larger focus on parent-training, compared to a treatment that placed more focus on children’s trauma-related memories and appraisals. Thus, characteristics of children’s posttrauma social environment may be more relevant to managing posttrauma externalising behaviours (Deblinger et al. 1996; Silverman et al. 2008).

Findings should be interpreted in the light of limitations. First, as this is an observational longitudinal study, causation cannot be determined. Second, we did not include a child-report measure of externalising symptoms in our study, and we were unable to compare exact measures of internalising (i.e., anxiety and depression) across child- versus parent-report. The approach we took to symptom measurement is consistent with best practice: parent or teacher reports are considered the gold standard for measuring children’s externalising (Stanger and Lewis 1993), whereas child-report is recommended for measurement of PTSS (e.g., Kassam-Adams et al. 2006; Meiser-Stedman et al. 2007b). Our observation that posttrauma child internalising problems reduced from 1-month to 6-months according to child-report but not parent-report may suggest that parents are reporting on children’s pre-established psychological profiles, rather than detecting changes in their posttrauma mental health. This is consistent with the view that parents may be relatively poor at detecting children’s posttrauma symptoms. Parental perceptions of their child’s distress may also be influenced by their own posttrauma distress. Nonetheless, single-informant bias may substantially inflate associations in the context of psychopathology, and associations between child-reported symptoms and cognitions should be considered with this in mind. Null findings should also be considered in the light of the relatively modest overall sample size. The sample size also meant we were not appropriately powered to run more sophisticated analyses, such as structural equation models, which may be better able to account for covariance between the different symptom outcomes. Finally, it is notable that, as is typical of low-risk acute-incident emergency department samples, overall internalising and externalising symptoms in our sample were relatively low. For the majority of participants the index trauma was an acute accidental injury, and while families were from a range of socio-economic backgrounds, the sample was mostly culturally- and ethnically-homogenous, and captured children up to 13 years old. Findings cannot be generalised to more complex traumas or high-risk groups, or to adolescents or other cultural contexts.

In conclusion, cognitive processes that are known to be important in the maintenance of PTSD are also likely to be relevant for children’s broader posttrauma internalising difficulties. Our findings particularly highlight the role of maladaptive coping and maladaptive appraisals, in predicting acute distress and reductions in non-PTSD internalising problems, and suggest that it may be beneficial to take a trauma-informed approach to addressing posttrauma internalising problems by targeting these processes. What remains to be seen is whether trauma informed treatment frameworks may effectively target trauma-related anxiety and depression in the absence of clinical-level PTSD.

## Electronic supplementary material

ESM 1 (DOCX 35 kb)
