# Supplementary material for: A Longitudinal Examination of the Relationship between Trauma-Related Cognitive Factors and Internalising and Externalising Psychopathology in Physically Injured Children
Source: J Abnorm Child Psychol. 2018 Sep 28;47(4):683–93. doi: 10.1007/s10802-018-0477-8 (PMC6439173; doi:10.1007/s10802-018-0477-8)
Supplement: Supplementary file 1 — (DOCX 35 kb) [file 10802_2018_477_MOESM1_ESM.docx]

S1. *Flow chart of recruitment numbers from* [removed for blinding]

341 eligible families

6-month follow-up: *n* = 127

(96% of original sample)

- 2 families were unable to be contacted

- 3 families were no longer interested in

participating

3-month follow-up: *n* = 111

(84% of original sample)

Primary reason for non-completion of 3-month follow-up was that questionnaires were not completed within the timeframe (no later than 3-months post-T1 (±1mo)

Reasons for non-contact:

- 94 could not be contacted within 1-month

- 52 were not interested/too busy

- 2 believed it would be too distressing for

child

Drop-out primarily because appointment was cancelled and was unable to be rescheduled within required 1-month post-hospital timeframe.

Final Sample at T1: *N* = 132

Reasons for not participating

- 25 could not be contacted or have their

first assessment scheduled within 1-month

- 13 were not interested/too busy

- 5 believed it would be too distressing for

child

151 agreed to participate

194 agreed to be contacted by research team
